# Oral isotretinoin for the treatment of chronic pityriasis versicolor: case report and literature review^☆^

**DOI:** 10.1016/j.abd.2023.08.014

**Published:** 2024-04-25

**Authors:** John Verrinder Veasey, Gustavo de Sá Menezes Carvalho, Guilherme Camargo Julio Valinoto

**Affiliations:** a Dermatology Clinic, Hospital da Santa Casa de São Paulo, São Paulo, SP, Brazil; b Discipline of Dermatology, Santa Casa de São Paulo School of Medical Sciences, São Paulo, SP, Brazil

Dear Editor,

Pityriasis versicolor (PV) is a superficial mycosis caused by yeast species of the *Malassezia* genus. It affects individuals worldwide, mostly in countries with tropical and subtropical climates, where the incidence can reach up to 50% of the population.^1^ Propaedeutic and complementary methods such as the Zirelí sign, dermoscopy, and Wood's light are useful both in the diagnosis of the infection and in evaluating the therapeutic response by helping to differentiate between active disease and post-inflammatory hypochromia.^1^, ^2^, ^3^, ^4^

Treatment is especially challenging in cases of recurrent or chronic evolution, not due to the pathogen intrinsic resistance to antifungals used topically and systemically, but due to individual host factors present in the human microbiome that favor an environment suitable for the persistence of the lipophilic fungus.^3^, ^5^ Scientific studies on the use of oral isotretinoin as an alternative to treat infection are scarce, the vast majority of which are case reports.^6^, ^7^, ^8^ The authors report on a patient with chronic PV without improvement following classical treatments based on antifungals, he showed a good response after a few weeks of oral isotretinoin use.

A 40-year-old healthy male patient, undergoing dermatological follow-up for eight years due to chronic PV was evaluated. During the period, he had undergone several treatments with antiseborrheic soaps, topical antifungals in shampoo, cream and spray lotion formulations (ciclopirox olamine and azole derivatives), in addition to oral treatments with ketoconazole, itraconazole and fluconazole, with little improvement and always maintaining a positive Zirelí sign, compatible with active infection.^2^, ^4^ Dermatological examination revealed diffusely oily skin and PV in a follicular pattern. The patient denied using creams and emollients, and the habit of taking two showers a day. Considering the chronic condition with resistance to classic therapies and oily skin characteristics, general laboratory tests were carried out, which showed normal results. Then an off-label treatment of oral isotretinoin as monotherapy was proposed, without association with previously reported treatments. A dose of 20 mg/week was prescribed, and the patient improved significantly after eight weeks of follow-up, without any laboratory alterations. A total dose of 160 mg was administered during the period (Figure 1, Figure 2, Figure 3). To improve the oily skin condition and prevent the recurrence of the infection, continuous use of this dose of isotretinoin was maintained.

**Figure 1:**
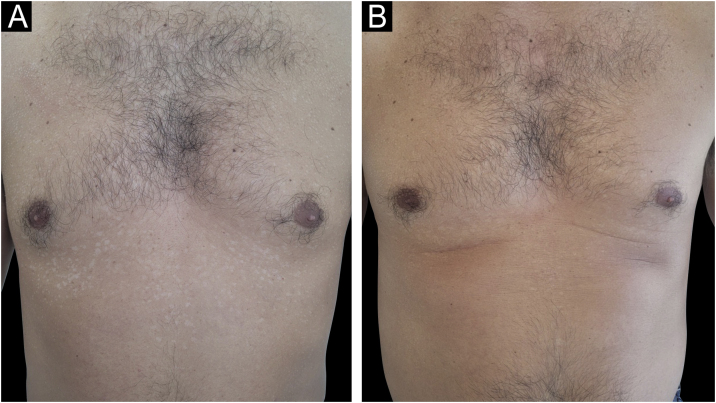
Patient with chronic pityriasis versicolor. (A) Before treatment. (B) After eight weeks of treatment with a low/weekly dose of oral isotretinoin.

**Figure 2:**
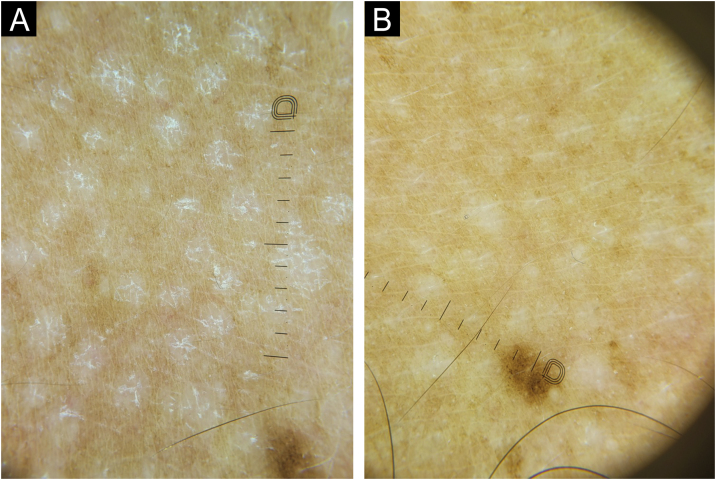
Dermoscopy with polarized light of a patient with chronic pityriasis versicolor. (A) Circular macules centered on the hair follicles, with the presence of desquamation after the Zirelí propaedeutic maneuver, compatible with active disease. (B) After eight weeks of treatment with oral isotretinoin, a reduction in the number and size of lesions was observed, with no desquamation after the Zirelí maneuver, compatible with post-inflammatory hypochromia after resolution (Dermatoscope DL5 Dermlite®, ×10).

**Figure 3:**
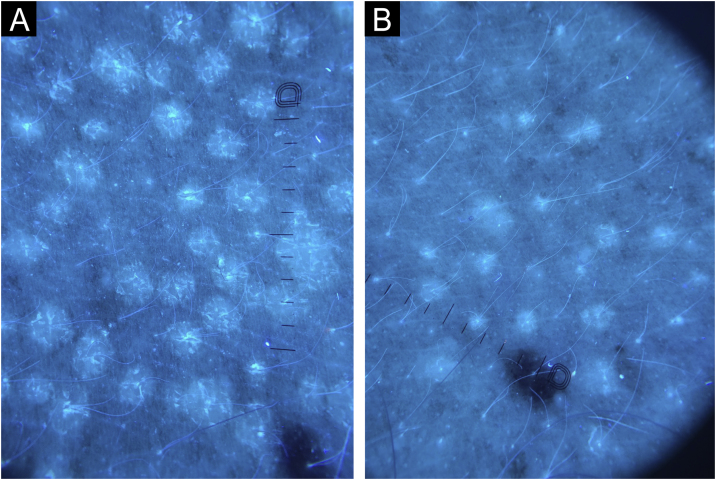
Dermoscopy with Wood's light (×10) in a patient with chronic pityriasis versicolor. Hypochromia and pre-treatment desquamation compatible with active disease are more accurately observed (A); and post-treatment with a reduction in the number and size of macules, in addition to absence of desquamation, compatible with post-inflammatory hypochromia after resolution (B). (Dermatoscope DL5 Dermlite®, ×10).

The classification of recurrent and chronic PV is based on the number of lesion recurrences after treatment with adequate antifungals over a two-year period: up to four for recurrent, and more than four for chronic conditions. As concluded by Framil et al., there is no correlation between the *Malassezia* species, the clinical form, and recurrence episodes, but a close relationship with individual predisposing factors.^3^

The use of isotretinoin for PV was initially described in 2006 by Bartel et al., in a case of a 14-year-old adolescent with PV on the back and severe acne, treated with 40 mg of isotretinoin, twice a day (1 mg/kg/ day), for five months. There was clinical and mycological cure of PV, suggesting a direct role against *Malassezia* or through the reduction of the cutaneous lipid content by the drug, making the environment less favorable for the lipophilic fungus.^6^ In 2018, Yazici et al. described another case of a patient with chronic PV for 15 years who was treated with oral isotretinoin at a dose of 20 mg/day for two months, showing sustained improvement after one year of medication withdrawal.^7^ In a recent extensive review study on isotretinoin, Bagatin et al. discuss several off-label uses of the drug, including seborrheic dermatitis but do not specifically address PV.^8^ Geissler et al. reported that treatment with very low doses of isotretinoin (2.5 mg/3 times a week) is effective in controlling seborrhea, with a 51% reduction in the size of the sebaceous gland, as demonstrated on histopathology.^9^

Reduced doses to control moderate acne have been successfully described since the 90 s,^10^ an opinion supported to this day by reviews that demonstrate a tendency to prescribe lower daily doses (0.1 − 0.5 mg/kg, up to 5 mg), for a longer period (up to 18 months), fewer adverse events, better tolerability and relapse rates similar to those observed with conventional doses.^8^

Although the efficacy of isotretinoin is well-known in acne, literature on its use in PV is scarce. The likely mechanism underlying the long-term efficacy and remission of PV with isotretinoin use seems to be related to decreased sebum production and sebaceous gland atrophy. Further studies are needed to establish the ideal dose and duration of therapy. The authors think that low-dose isotretinoin may be a good therapeutic option for recurrent and chronic PV. It is clear that research into the use of isotretinoin to control PV is a field that is extremely lacking in robust and consistent studies.

## Financial support

None declared.

## Authors’ contributions

John Verrinder Veasey: Design and planning of the study; data collection, or analysis and interpretation of data; drafting and editing of the manuscript or critical review of important intellectual content; collection, analysis and interpretation of data; intellectual participation in the propaedeutic and/or therapeutic conduct of the studied cases; critical review of the literature; approval of the final version of the manuscript.

Gustavo de Sá Menezes Carvalho: Design and planning of the study; data collection, or analysis and interpretation of data; drafting and editing of the manuscript or critical review of important intellectual content; collection, analysis and interpretation of data; critical review of the literature; approval of the final version of the manuscript.

Guilherme Camargo Julio Valinoto: Design and planning of the study; data collection, or analysis and interpretation of data; drafting and editing of the manuscript or critical review of important intellectual content; collection, analysis and interpretation of data; critical review of the literature; approval of the final version of the manuscript.

## Conflicts of interest

None declared.
